# Towards High-Performance Inverted Mesoporous Perovskite Solar Cell by Using Bathocuproine (BCP)

**DOI:** 10.3390/molecules29174009

**Published:** 2024-08-24

**Authors:** Yongjun Wei, Feiping Lu, Xinqi Ai, Ju Lei, Yong Bai, Zhiang Wei, Ziyin Chen

**Affiliations:** 1 Engineering Research Center of Integrated Circuit Packaging and Testing, Ministry of Education, Tianshui Normal University, Tianshui 741000, China; a15588277079@163.com (Y.W.); 15174580654@163.com (X.A.); lj15996312828@163.com (J.L.); by2016fighting@163.com (Y.B.); 18724672618@163.com (Z.W.); 18225101250@163.com (Z.C.); 2 Department of Microelectronics, Tianshui Normal University, Tianshui 741001, China

**Keywords:** perovskite solar cell, buried interface, mesoporous structure, lead chelator, photoelectric conversion efficiency

## Abstract

Perovskite solar cells (PSCs) are considered the most promising photovoltaic devices to replace silicon-based solar cells because of their low preparation cost and high photoelectric conversion efficiency (PCE). Reducing defects in perovskite films is an effective means to improve the efficiency of PSCs. In this paper, a lead chelator was selected and mixed into hole transport layers (HTLs) to design and prepare mesoporous PSCs with the structure of ITO/PTAA(BCP)/Al_2_O_3_/PVK/PCBM/BCP/Ag, and its modification effect on the buried interface at the bottom of the perovskite layer in the mesoporous structure was explored. The experimental results show that in the presence of mesoporous alumina, the lead chelator can still play a role in modifying the bottom of the perovskite film. The use of lead chelator as passivation material added to the HTL can effectively reduce the residue of dimethyl sulfoxide (DMSO) and decrease the defects at the bottom of the perovskite film, which dramatically improves the device performance. The PCE of the device is increased from 18.03% to 20.78%, which is an increase of 15%. The work in this paper provides an effective method to enhance the performance of PSCs.

## 1. Introduction

The application of green renewable energy can effectively alleviate the social problems caused by fossil fuels. Among them, the development of the photovoltaic industry is currently one of the effective ways to solve the problems of environmental pollution and energy shortage [1,2,3]. At present, the utilization of solar energy mainly relies on silicon-based solar cells, but it is difficult to improve the production cost of silicon-based solar cells due to the high cost of production, and the maximum conversion efficiency is limited by the Shockley–Queisser limit [4,5,6]. Therefore, we need better photovoltaic devices. Perovskite solar cells (PSCs) have attracted the attention of various researchers due to their advantages of low preparation cost and high photoelectric conversion efficiency (PCE). Since the first report of PSCs, the PCE of single-cell PSCs has exceeded 26.1% [7]. Improving the PCE and stability of PSCs is still the focus of current research and hot issues, while defects in perovskite films are one of the main factors affecting device efficiency and stability.

Currently, commonly used approaches to reduce the defects in the perovskite layer are improving the crystalline quality of perovskite films and surface passivation [8,9,10,11,12]. In terms of reducing the defects in perovskite films, researchers tend to focus on the top of the perovskite film because it is more convenient to observe the top morphology and properties than that at the bottom, so there are relatively few research works on the bottom of the perovskite film, but non-radiative recombination of interfacial carriers induced by buried interfacial defects at the bottom may limit the efficiency of the p-i-n-structured PSCs [13]. Recently, some research groups have started to focus on the optimization of the bottom buried interface. Yang et al. invented a nondestructive stripping technique for perovskite polycrystalline films by dissolving the bottom polymer transport layer with chlorobenzene immersion, stripping the perovskite film from the transparent substrate, and obtaining the bottom of the perovskite polycrystalline film with complete exposure for the first time, and they found that the heterogeneity at the bottom of the perovskite film was much higher than that at the top surface, the inhomogeneity of lead halide at the bottom interface is an important cause of the defects at the bottom, and they found that passivation by ammonium halide can optimize the buried bottom interface from top to bottom, thus enhancing the crystalline quality of the whole film [14]. Luo et al. chose to uncover the perovskite films with UV adhesive to expose the buried bottom interface, and they found that the disorder of the primary stage of the growth of perovskite film can deteriorate the buried interface. Instead of using additives or passivators, they synthesized transparent conductive oxide perovskite (SrSnO_3_) as an electron transport layer (ETL); this ETL material led to a more ordered growth of the bottom lattice, which improved the bottom crystallinity and reduced the generation of voids at the bottom [15]. Zhang et al. designed a series of guanidinium salts with varying degrees of amino substitution as modifiers. Through calculations, they found that the main factor affecting the effect of the bottom modification is the binding ability of the modifier to the ETL, which is a way to reduce the defects at the bottom of the perovskite layer by passivating the ETL at the bottom of the perovskite layer [16]. Zhang et al. proposed to use a starch-multimode supramolecule as a bifunctional buffer layer, which is introduced at the perovskite interface to the buried interface and to regulate the iodide ion migration by starch-I, thereby reducing the iodine vacancy defects. The modified PSCs achieved high efficiency and stability [17]. Chen et al. found that dimethyl sulfoxide (DMSO), an additive commonly used in perovskite films, led to voids at the perovskite substrate due to its easy capture in the early stage of the film-forming process, resulting in a film that is prone to degradation in the light, and they chose to partially replace the DMSO with carbonyloxyhydrazide (CBH) as a way to reduce the DMSO residue [18].

During the film formation process of the perovskite films, DMSO combines with lead ions at the bottom to form a stabilized phase; therefore, the evaporation of DMSO forms voids at the bottom on the scale of tens of nanometers [19]. There is a strong interaction between the lead chelator and the lead ions; therefore, when the two are in contact, the lead chelator will bind with the lead ions first. We chose bathocuproine (BCP) as the lead chelating agent. After the addition of BCP, DMSO will evaporate in the early stage of film formation due to the lack of ligand Pb^2+^, which reduces the residue of DMSO, thus reducing the bottom defects [19]. N,N-Dimethylformamide (DMF) is a common solvent for the solution of perovskite precursors, and due to the high solubility of DMF [20], the addition of a small amount of BCP is easily thrown out during the spin-coating process. To solve this problem, we chose to add BCP in the bottom cavity transport layer, but the mesoporous structure tends to spin-coat the bottom of the perovskite layer as a way to increase the wettability of the perovskite layer, and the modification of the interface at the bottom may not be able to play a very good role due to the existence of the mesoporous layer. Since the mesoporous structure is often closely connected to the bottom of the perovskite film, and the particles of the mesoporous structure, such as alumina, are often large, the large particles of alumina may block the contact between the HTL and the perovskite layer. 

We experimentally found that, in the PSC with mesoporous structure, the bottom of the perovskite layer can still be passivated by placing an appropriate amount of BCP in the bottom of the HTL, and the open-circuit voltage (V_OC_) of the device was greatly improved from the maximum of 1.15 V to 1.18 V. Meanwhile, the short-circuit current density (J_SC_) and filling factor (FF) were improved to a certain extent, and PCE was increased from 18.03% to 20.78%. The introduction of lead chelating agents greatly reduces the bottom defects of mesoporous PSCs and decreases nonradiative recombination, resulting in significantly improved PCE.

## 2. Results and Discussion

**Figure 1:**
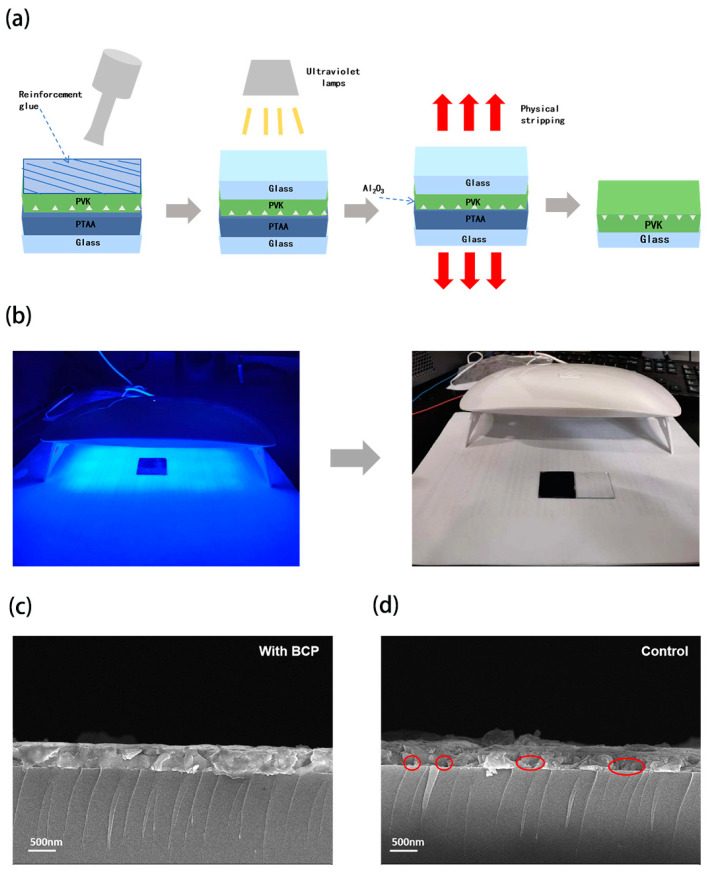
Schematic diagram of nondestructive physical stripping of perovskite film (**a**), the irradiation process of two pieces of glass under UV lamp and the final peeling effect (**b**), cross-sectional SEM image of PSCs (**c**) for the experimental group, and (**d**) for the control group.

It is well known that for inverted PSCs where the perovskite film is grown on an HTL, the quality of the buried interface is particularly important for the crystallization quality of the perovskite crystals, and the crystallization state of the starting bottom buried interface also determines to a certain extent the crystallization quality of the top perovskite film, which affects the crystallization of the whole film [14]. Lead chelating agent BCP is also a Lewis base in nature and has been reported to bind to uncoordinated Pb^2+^ to reduce PbI_2_ content in some literature [21,22]. However, a small amount of PbI_2_ has been reported in the literature to be beneficial to the work of PSCs [23], and our BCP additions are small, so we will not discuss PbI_2_ for the moment. To explore the crystallization of the bottom buried interface of the perovskite film, we tried to use a common nail reinforcement glue to make the bottom exposed, as shown in Figure 1a,b. Because the contact between the PTAA/PVK interfaces is not strong, we prepared the structure of the ITO/PTAA/Al_2_O_3_/PVK device and uniformly applied the glue to the top of the perovskite film, and then quickly pasted onto another clean glass sheet. After UV lamp irradiation for a period of time, we broke it to obtain a complete buried interface. We chose BCP as the passivator, which was proven to be an effective Pb chelator for perovskite films in other articles [16]. Then, we doped some BCP in small quantities and prepared the structures of ITO/PTAA(BCP)/Al_2_O_3_/PVK and ITO/PTAA/Al_2_O_3_/PVK devices, which was investigated by SEM. As shown in Figure 1c,d, it can be observed that there are many voids marked in red circles on the cross-sectional images of the control group, which can seriously affect the performance of the device. The devices with addition of BCP show significantly reduced gaps at the bottom of the perovskite film due to the reduction in residual DMSO, resulting in a more compact bottom arrangement and superior morphology. This suggests that BCP can effectively passivate the interface at the bottom of perovskite films, reduce the generation of voids, decrease the defects at the bottom, and optimize the performance of the device.

**Figure 2:**
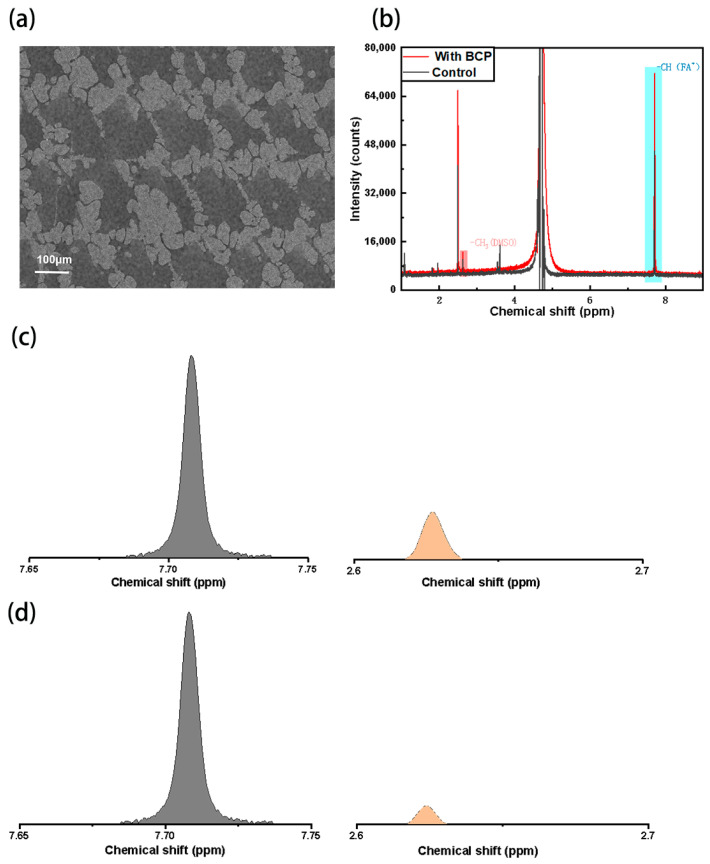
SEM images of the bottom of the perovskite film at 100 μm scale (**a**), and ^1^H-NMR results of the experimental and control groups (**b**), schematic area integration of the ^1^H-NMR results of the control group (**c**), and the area integration of the ^1^H-NMR results of the experimental group (**d**).

Subsequently, we observed the bottom of the perovskite film using a SEM, as shown in Figure 2a. It is found that the Al_2_O_3_ particles were distributed at the bottom of the perovskite film in the form of “islands”, which was similar to the results obtained by Xu et al. [24]. Therefore, the presence of Al_2_O_3_ particles did not completely block the contact between the perovskite layer and the HTL. Because of contact with the cavity transport layer, we speculate that the addition of Pb chelator to the cavity transport layer can still achieve the effect of reducing the DMSO residue and optimizing the bottom interface. In order to prove that the addition of BCP can reduce the residual DMSO in the perovskite film, we prepared the samples with BCP and the control samples on the glass sheet, which were scraped off with a blade and melted into D_2_O for hydrogen nuclear magnetic resonance spectroscopy test, as shown in Figure 2b. Since the residual amount of each group on the glass sheet is different after the film is scraped off with a blade, only the level of DMSO(-CH_3_) characteristic peak in the hydrogen ^1^H-NMR spectrum cannot accurately reflect the content of DMSO in the perovskite film, but the introduction of BCP will not affect the content of FA^+^. Therefore, we took the integral area ratio of the characteristic peaks of FA^+^(-CH) and DMSO(-CH_3_) as the standard to measure the content of DMSO. As shown in Figure 2c,d, the characteristic peaks of FA^+^(-CH) and DMSO(-CH_3_) were 7.71 ppm and 2.63 ppm, respectively. We used the integral area ratio of characteristic peaks to represent the content of DMSO in perovskite film, and calculated that the content of DMSO in the experimental group was 0.43%, and that in the control group was 0.72%. It can be seen that the proportion of DMSO in the experimental system is less than that in the control group, so the samples added with BCP can still compete with DMSO in Pb^2+^ when blocked by Al_2_O_3_ nanoparticles, thereby reducing the residue of DMSO.

**Figure 3:**
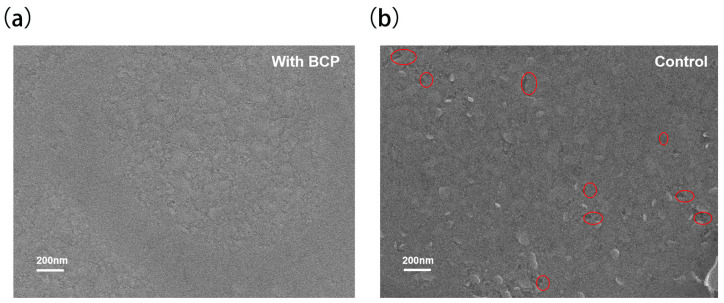
SEM images of the bottom of the perovskite film at 200 nm scale for the experimental group (**a**), and the control group at 200 nm scale for the control group (**b**).

In order to verify that the reduction in DMSO can improve the bottom morphology of perovskite films with mesoporous structure, PSCs with the structure of ITO/PTAA(BCP)/Al_2_O_3_/PVK and ITO/PTAA/Al_2_O_3_/PVK devices were carefully designed and crafted, which were completely exfoliated. SEM images were taken, as shown in Figure 3a,b, which showed that the samples with the addition of BCP can modify the bottom of the perovskite. Good morphology is beneficial to the crystallization quality of perovskite films. This reduces the voids and forms a dense and non-porous bottom, while the control sample without BCP has significantly more voids and more defects.

**Figure 4:**
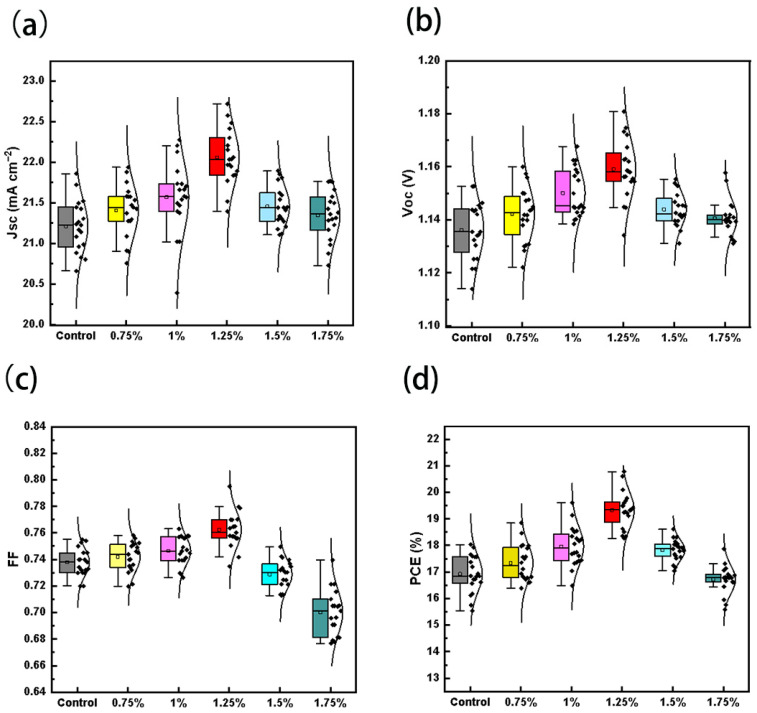
Statistical results of (**a**) J_SC_, (**b**) V_OC_, (**c**) FF, and (**d**) PCE with different loading ratio.

In order to investigate at which concentration of BCP can maximally improve the performance of the PSCs, we prepared BCP with mass ratios of 0%, 0.75%, 1%, 1.25%, 1.5%, and 1.75% for the concentration test, and in order to prove the reproducibility of the experiments, we prepared 20 devices in each group plotted as box line diagrams, as shown in Figure 4 and Table 1. The control group has the highest PCE of 18.03% and the average PCE of 16.93% due to more defects at the bottom, and its various performances are improved with the increase in the doping ratio of BCP. It reaches its highest efficiency at the concentration of 1.25% by mass ratio, and the short-circuit current, the open-circuit voltage, the filling factor, and the photoelectric conversion efficiency of the cells are all improved. The highest PCE reached 20.78%, and the average PCE was also 19.33%. It is noteworthy that the doping of BCP led to a significant increase in the open-circuit voltage, which may be due to the improvement of the perovskite film [25]. Due to the excellent hole-blocking ability of BCP, as it is added in the HTL, if the concentration of doped BCP exceeds 1.25%, it may block the hole transport, which may have a bad effect on the performance of the device [26,27].

**Figure 5:**
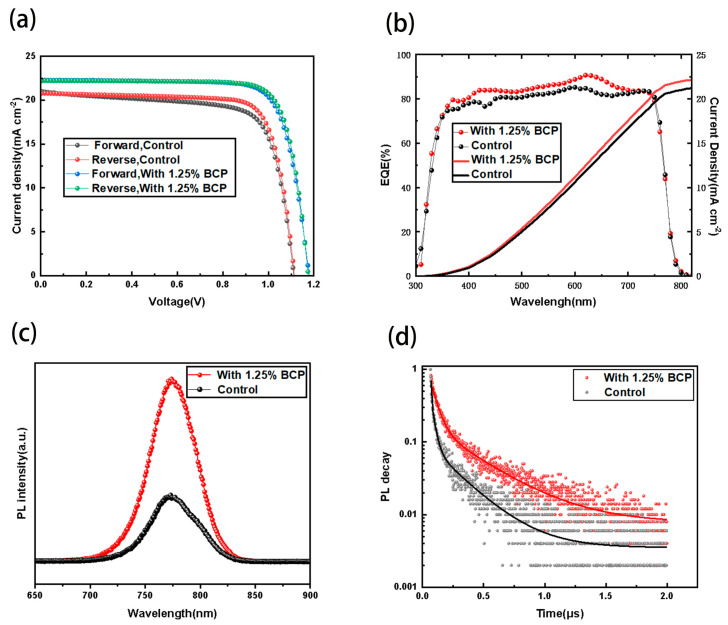
Forward and backward sweep curves of control and optimal BCP concentration devices (**a**), EQE of control and optimal BCP concentration devices (**b**), static PL spectra of control and optimal BCP concentration devices (**c**), transient PL spectra of control and optimal BCP concentration devices (**d**).

Subsequently, we performed forward and backward sweep tests on the devices at the optimal concentration, as shown in Figure 5a. The J-V curves of the forward and backward sweeps of the devices with the addition of BCP almost overlapped, and the details of the forward and backward sweeps as well as their respective hysteresis are shown in Table 2, which was 5.5% for the control group, while the hysteresis of the devices with the addition of optimal concentration of BCP was 1.6%, which indicated that the addition of BCP led to a reduction in defects in the PSCs, and the ion mobility is reduced and non-radiative recombination is significantly reduced, improving the performance and stability of the devices [28,29,30]. The results of the external quantum efficiency (EQE) test of the devices are shown in Figure 5b, and the spectral response of the devices with added BCP is enhanced in the range of 350 nm–750 nm, with the highest quantum efficiency reaching 91%, compared to only 85% for the control group. The integral currents obtained were 22.13 mA/cm^2^ and 21.20 mA/cm^2^ for the optimal BCP concentration device and control device, respectively, which are approximately the same with the average current density. The short-circuit currents of the calomel solar cells were also improved due to the optimization of the bottom defects [31]. In order to investigate the effect of BCP addition on the defects in the perovskite layer of the device, we prepared ITO/PTAA(BCP)/Al_2_O_3_/PVK and ITO/PTAA/Al_2_O_3_/PVK devices and measured the steady-state PL and transient PL life tests of the devices as shown in Figure 5c,d. The BCP-added devices had higher PL intensity, indicating the smaller proportion of non-radiative recombination, the fewer defects in the perovskite film, and the better crystallization condition of the light-absorbing layer, which is conducive to carrier transport. In contrast, the PL intensity of the control group was lower, suggesting that the device without BCP has more defects and more voids at the bottom of the film in the perovskite film, which is unfavorable for carrier transport.

Transient PL spectra are commonly used to characterize the charge complexation and charge extraction capabilities within and at the interfaces of the films, and the collected data can be fitted by the following equation:

(1) $$I(t)=A_{1}\exp(-t/\tau_{1})+A_{2}\exp(-t/\tau_{2})+y_{0}$$

where *A*_2_ and *A*_1_ are relative amplitudes (m), and *τ_1_* and *τ_2_* are fast decay lifetimes and slow decay lifetimes, respectively. It is calculated that the addition of BCP increases the average carrier lifetime *τ_ave_* of perovskite thin films from 75 ns to 178 ns, and the specific data are presented in Table 3. This indicates that the addition of BCP enhances the bottom crystallization of the perovskite layer film, generates more electron-hole pairs, reduces the non-radiative recombination center, and improves the carrier transport capacity [32,33,34].

**Figure 6:**
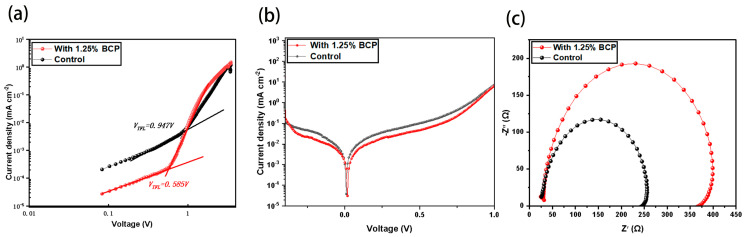
SCLC test for control and optimal BCP concentration devices (**a**), dark state current test for both control and optimal BCP concentration devices (**b**), and EIS test for control and optimal BCP concentration devices (**c**).

To further verify the passivation effect of BCP on perovskites, we adopted the space charge limiting current method (SCLC) to measure the defect state density of perovskite films, as shown in Figure 6a. We prepared single carrier (hole) devices with structures ITO/PTAA(BCP)/Al_2_O_3_/PVK/PTAA/Ag and ITO/PTAA/Al_2_O_3_/PVK/PTAA/Ag, and the J-V curves of the devices were measured in the dark state. The relationship between the density of defect states (N_traps_) and the V_TFL_ can be expressed by following equation [35]:

(2) $$N_{traps}=2\varepsilon \varepsilon_{0}V_{TFL}/eL^{2}$$

The inflection point *V_TFL_* in the figure is the trap limit filling voltage, *e* is the elementary charge, *ε* is the relative permittivity of perovskite (*ε* = 32), *ε_0_* is the vacuum permittivity, and *L* is 450 nm. The calculation results show that the defect state density after the addition of BCP decreases from 1.65 × 10^16^ cm^−3^ to 1.02 × 10^16^ cm^−3^. The decrease in the defect state density reflects the fact that the addition of BCP makes the defects in the perovskite film reduced, and also verifies the previous test results of PL spectra and transient emission spectra [36,37,38].We performed J-V dark current tests under light-free conditions for the control and experimental groups, respectively, and as shown in Figure 6b, the reverse leakage current density of the device was significantly reduced after the addition of BCP, implying that the nonradiative compounding was significantly suppressed. In addition, we tested the electrochemical impedance spectra under light-free conditions, as shown in Figure 6c, and the electrochemical impedance spectroscopy (EIS) results show that the composite resistance R_rec_ of the control group is 245 Ω, and the composite resistance R_rec_ of the BCP-modified device is 368 Ω. The significant increase in the recombination resistance R_rec_ means that the recombination rate of electrons and holes in the PSCs is reduced, which means that more photo-generated carriers can participate in the transmission of current, rather than recombination in vivo or at the interface, further demonstrating that the film quality of the device is improved after the addition of BCP [39].

## 3. Materials and Methods

**Figure 7:**
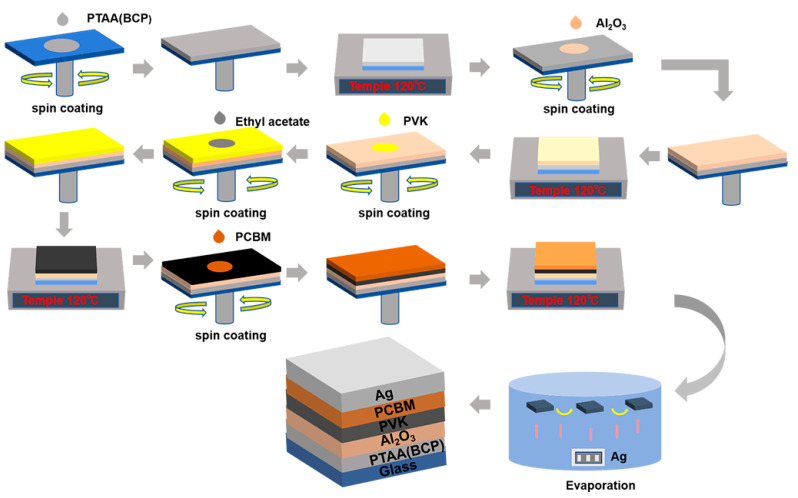
Schematic diagram of device fabrication process and device structure.

The solvents used in this work including isopropanol, chlorobenzene, DMSO, DMF, EA were purchased from Aladdin with 99.9% purity (Tokyo, Japan). Al_2_O_3_ was purchased from Sigma-Aldrich (St. Louis, MO, USA) and poly[bis(4-phenyl) (2,4,6-trimethylphenyl) amine (PTAA), CsI, MABr, PbBr_2_, FAI, PbI_2_, Phenyl C61 Butyric Acid Methyl Ester (PCBM), BCP, and 2-Phenylethylamine Hydroiodide (PEAI) were purchased from Paulette (London, UK), all at 99.9% concentration. Nail reinforcement glue was purchased from Perfect Color. Figure 7 shows the device preparation flow in this experiment. We used a mask with an aperture area of 0.049 cm^2^. Firstly, we cleaned the ITO glass sheet with detergent, deionized water, and ethanol in a KQ-50E ultrasonic cleaner for 15 min, and then baked it in an electric blast drying oven at 70 °C for 2 h. It was cleaned with a UV light cleaner for 10 min before spin-coating. FAI (247.4 mg) and PbI_2_ (691.5 mg) were dissolved in 1 mL DMF:DMSO = 4:1 solvent mixture, 154.1 mg of MABr and 550.5 mg of PbBr_2_ in 1 mL of a 4:1 solution, and CsI in DMSO at a concentration of 390 mg/mL. The above three solutions were heated at 60 °C for 1 h to dissolve completely and then mixed in different ratios to obtain the precursor solution. The chemical formula was Cs_0.05_FA_0.83_MA_0.12_PbI_2.64_Br_0.36_. BCP was doped into the PTAA at a PTAA mass ratio of (0%, 0.75%, 1%, 1.25%, 1.5%, 1.75%) solution, 200 μL of 1.5 mg/mL PTAA chlorobenzene solution was taken and spin-coated on ITO glass at 6000 rpm, followed by annealing at 120 °C for 20 min. After cooling for three minutes, the Al_2_O_3_ suspension was mixed with isopropanol and spin-coated on the substrate at 3500 rpm for 30 s, and annealed at 120 °C for 20 min. Then, the prepared perovskite precursor solution was spin-coated at 4000 rpm for 30 s, and EA was used as the anti-solvent during the spin-coating process, and the glass slice was quickly transferred and annealed at 100 °C for 20 min. An amount of 1 mg/mL of PEAI isopropanol solution was then spin-coated at 4000 rpm for 30 s, and then annealed at 100 °C for 10 min. An amount of 15 mg/mL of PCBM chlorobenzene solution was then spin-coated at 2000 rpm for 30 s. The supernatant of BCP was spin-coated at 4000 rpm for 30 s. Finally, the electrode Ag was vaporized.

Scanning electron microscopy (SEM): The bottom image of a 200 nm sized perovskite film and the cross-sectional image of a 500 nm sized perovskite film were captured under TESCAN MIRA LMS (TESCAN, Brno, Czechia). For SEM characterization, the electron beam accelerating voltage was 3 KV and the current was 100 pA, and the SEM images at 100 μm size were taken under SU3800 (a SEM instrument model, Hitachi High-Tech, Tokyo, Japan). The electron beam accelerating voltage was 5 KV, and the current was 86 pA. When preparing the sample, nail reinforcement glue was applied on a blank glass sheet, and then the prepared glass/PTAA/Al_2_O_3_/PVK sample was pressed on the glass sheet covered with nail reinforcement glue. The bubbles were squeezed out, a UV lamp was shined on the sheet for 15min, and it was physically broken apart to take the peel off.

^1^H-NMR spectra: ^1^H-NMR spectra were obtained using a Bruker AVANCE III 600 MHz ^1^H-NMR spectrometer (Bruker Corporation, Braunschweig, Germany). The glass/PTAA/Al_2_O_3_/PVK samples were first prepared on a glass sheet, and the layers of film on the samples were scraped off with a razor blade and poured into D_2_O, which was put into the ^1^H-NMR tube.

Steady-state photoluminescence spectroscopy (PL) and time-resolved photoluminescence spectroscopy (TR-PL) spectra: PL spectra were obtained using Hitachi F-4600 fluorescence spectrophotometer (Hitachi, Tokyo, Japan), and time-resolved photoluminescence (TR-PL) spectra were obtained using FLS980 (Edinburgh Instruments, Livingston, UK) under excitation light at a wavelength of 450 nm.

External Quantum Efficiency (EQE): The complete devices were prepared to measure the EQE of the devices using a spectral correspondence system (Enlitech QE-R, Enlitech, Taiwan, China), and the spectral response was calibrated using the silicon solar cell as a reference. 

The complete equipment was prepared by electrochemical workstation Zahner XML (ZAHNER-GIMPS, Kroppenstedt, Germany) and tested by electrochemical impedance spectroscopy.

## 4. Conclusions

In summary, we applied the lead chelator (BCP) to PSCs containing mesoporous nano-Al_2_O_3_, used the physical stripping method to strip the perovskite film from the glass sheet non-destructively, explored the morphology of the bottom of the mesoporous layer, and then carried out a variety of characterizations, which proved that the introduction of the BCP can indeed reduce the residual of DMSO and improve the mesoporous PSCs’ bottom topography, thus reducing the defects of the perovskite film and enhancing the efficiency of the PSC. Eventually, the open-circuit voltage was greatly increased compared with the control group, and the PCE reached 20.78%, which increased by 15% relative to the control group.

## Figures and Tables

**Table 1:** Statistical performance of devices.

BCP Doping Ration		J_SC_ (mA cm^−2^)	V_OC_ (V)	FF	PCE (%)
0	Average	21.22 ± 0.10	1.14 ± 0.00	0.74 ± 0.00	16.93 ± 0.48
	Best cell	21.86	1.15	0.755	18.03
0.75%	Average	21.41 ± 0.10	1.14 ± 0.00	0.74 ± 0.00	17.34 ± 0.47
	Best cell	21.94	1.16	0.758	18.85
1%	Average	21.57 ± 0.19	1.15 ± 0.00	0.75 ± 0.00	17.96 ± 0.54
	Best cell	22.27	1.17	0.763	19.61
1.25%	Average	22.06 ± 0.11	1.16 ± 0.00	0.76 ± 0.00	19.33 ± 0.48
	Best cell	22.72	1.18	0.80	20.78
1.5%	Average	21.46 ± 0.06	1.14 ± 0.00	0.73 ± 0.00	17.82 ± 0.16
	Best cell	21.89	1.16	0.75	18.61
1.75%	Average	21.35 ± 0.09	1.14 ± 0.00	0.70 ± 0.00	16.63 ± 0.43
	Best cell	21.77	1.16	0.74	17.88

**Table 2:** The details of the forward and reverse sweeps and their respective hysteresis.

Sample	Scan Direction	J_SC_ (mA cm^−2^)	V_OC_ (V)	FF	PCE (%)	Hysteresis Index (%)
With 1.25% BCP	Reverse	22.23	1.17	0.80	20.78	1.6
	Forward	22.30	1.17	0.78	20.45	
Control	Reverse	20.80	1.12	0.77	17.57	5.5
	Forward	21.03	1.11	0.73	16.61	

**Table 3:** TR-PL fitting parameters for different samples.

Sample	*A_1_*	*τ_1_* (ns)	*A_2_*	*τ_2_* (ns)	*τ_ave_* (ns)
With 1.25% BCP	1.9	52.9	0.2	367.8	178
Control	3.5	29.1	0.1	257.2	75

## Data Availability

The data that support the findings of this study are available from the corresponding author upon reasonable request.
